# Using Canvas for an Interprofessional Virtual Team Visit

**DOI:** 10.1093/geroni/igab046.403

**Published:** 2021-12-17

**Authors:** Ashley Reed, Jennifer Mendez

**Affiliations:** Wayne State University, Detroit, Michigan, United States

## Abstract

The is session will demonstrate how to use the Canvas learning management system (LMS) to organize and facilitate interprofessional education experiences (IPE) amongst students and faculty. Emphasis will be placed on the use modules as a way to organize content and facilitate requirements associated with IPE. In addition, the session will include demonstration on how to assign disciplines to sections to aide in faculty abilities to review of student submissions.

